# Supplementary material for: Melanometry for objective evaluation of skin pigmentation in pulse oximetry studies
Source: Commun Med (Lond). 2024 Jul 11;4:138. doi: 10.1038/s43856-024-00550-7 (PMC11239860; doi:10.1038/s43856-024-00550-7)
Supplement: Supplementary file 2 — Supplementary Information [file 43856_2024_550_MOESM2_ESM.pdf]

Supplementary Information

Melanometry for Objective Evaluation of Skin Pigmentation in Pulse Oximetry Studies

Sandhya Vasudevan, William C. Vogt, Sandy Weininger, and T. Joshua Pfefer
Center for Devices and Radiological Health, Food and Drug Administration, 10903 New Hampshire Ave, Silver Spring, MD 20993, USA

Supplementary Figure 1 Subjective skin color classification methods

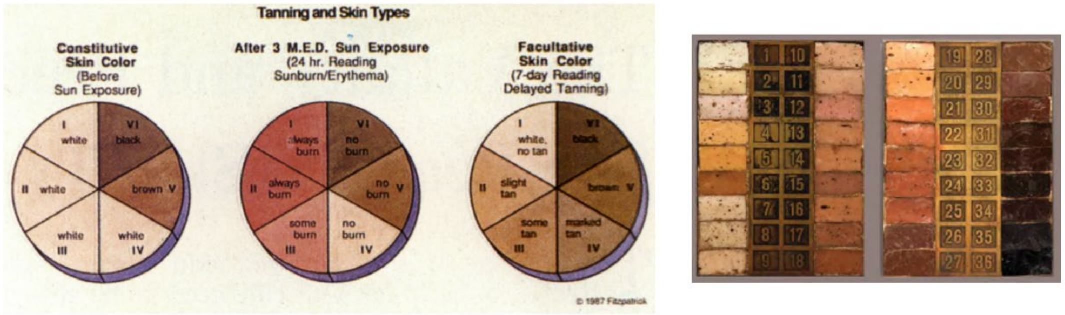

(a) Fitzpatrick skin phototype [1] (Reprinted with permission from American Medical Association) and (b) Von Luschan's chromatic scale [2] (Reprinted with permission from John Wiley and Sons)

Supplementary Figure 2 Crosstalk between melanometer melanin and erythema outputs

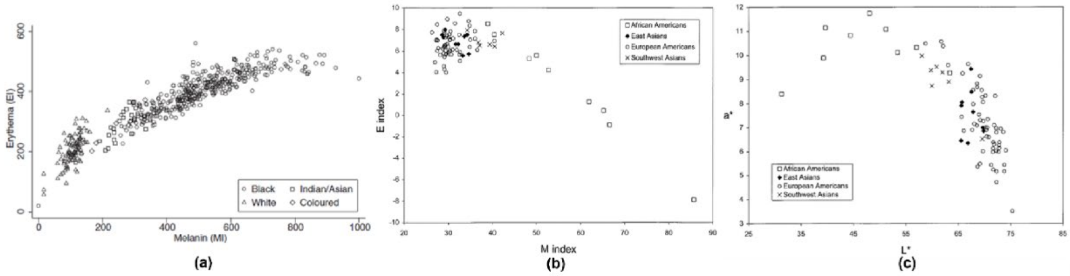

Crosstalk between melanin vs erythema outputs in (a) Mexameter MX18 [3] (Reprinted with permission from Photochem. Photobiol.), (b) Dermaspectrometer [4] and (c) L* vs a* for Photovolt [4]

References

1. T. B. Fitzpatrick, "The validity and practicality of sun-reactive skin types I through VI," Arch. Dermatol. **124**, 869-871 (1988).
2. A. K. Swiatoniowski, E. E. Quillen, M. D. Shriver, and N. G. Jablonski, "Technical note: comparing von Luschan skin color tiles and modern spectrophotometry for measuring human skin pigmentation," Am. J. Phys. Anthropol. **151**, 325-330 (2013).
3. C. Y. Wright, A. E. Karsten, M. Wilkes, A. Singh, J. du Plessis, P. N. Albers, and P. A. Karsten, "Diffuse reflectance spectroscopy versus Mexameter((R)) MX18 measurements of melanin and erythema in an African population," Photochem. Photobiol. **92**, 632-636 (2016).
4. M. D. Shriver, and E. J. Parra, "Comparison of narrow-band reflectance spectroscopy and tristimulus colorimetry for measurements of skin and hair color in persons of different biological ancestry," Am. J. Phys. Anthropol. **112**, 17-27 (2000).
