# Supplementary material for: Melanometry for objective evaluation of skin pigmentation in pulse oximetry studies
Source: Commun Med (Lond). 2024 Jul 11;4:138. doi: 10.1038/s43856-024-00550-7 (PMC11239860; doi:10.1038/s43856-024-00550-7)
Supplement: Supplementary file 3 — Description of additional supplementary files [file 43856_2024_550_MOESM3_ESM.pdf]

Description of Additional Supplementary Files

File name: Supplementary Data 1
Description: Expanded Summary of melanometer characteristics.

File name: Supplementary Data 2
Description: Compiled correlation results between commercial melanometers and FSP included in Figure 4 a.

File name: Supplementary Data 3
Description: Compiled correlation results between commercial melanometers included in Figure 5 a.

File name: Supplementary Data 4
Description: Repeatability results included in Figures 5 c-e.

File name: Supplementary Data 5
Description: Compiled correlation results of melanometer outputs to high quality melanin metrics included in Figure 6 g.
